# A review of cassava semolina (gari and eba) end‐user preferences and implications for varietal trait evaluation

**DOI:** 10.1111/ijfs.14867

**Published:** 2020-11-21

**Authors:** Wasiu Awoyale, Emmanuel Oladeji Alamu, Ugo Chijioke, Thierry Tran, Hubert Noel Takam Tchuente, Robert Ndjouenkeu, Ngoualem Kegah, Busie Maziya‐Dixon

**Affiliations:** ^1^ Food and Nutrition Sciences Laboratory International Institute of Tropical Agriculture PMB 5320 Oyo Road Ibadan Oyo State Nigeria; ^2^ Department of Food Science and Technology Kwara State University Malete PMB 1530 Ilorin Kwara State Nigeria; ^3^ Food and Nutrition Sciences Laboratory International Institute of Tropical Agriculture (IITA) Southern Africa Hub PO Box 310142, Chelstone Lusaka Zambia; ^4^ National Root Crops Research Institute, Umudike PMB 7006 Umuahia Abia State Nigeria; ^5^ Alliance of Bioversity International and the International Center for Tropical Agriculture (CIAT) CGIAR Research Program on Roots Tubers and Bananas (RTB) Apartado Aéreo 6713 Cali Colombia; ^6^ Qualisud CIRAD Montpellier SupAgro University of Avignon University of La Réunion University of Montpellier 73 rue JF Breton Montpellier 34398 France; ^7^ Department of Rural Socio‐Economics and Agricultural Extension Faculty of Agronomy and Agricultural Sciences University of Dschang PO Box 222 Dschang Cameroon; ^8^ International Institute of Tropical Agriculture PO Box 2008 Messa Yaoundé Cameroon

**Keywords:** Cassava root composition, gari and eba composition, sensory evaluation, texture and physicochemical analysis

## Abstract

The purpose of this review is to support breeders and food scientists by examining research carried out on end‐user preferences for gari and its derived dough product, eba, in Africa. The review focused on gari regarding the physical and chemical composition of raw cassava roots, methods of storage, the composition of gari with or without enrichment, and the sensory evaluation of gari and eba. The primary sensory attributes identified to describe gari are colour, taste, texture, aroma and flavour. Texture attribute of importance is crispiness for uncooked gari, and hand feel before consumption for eba. There was a significant correlation between the sensory characteristics of gari and the starch and cyanogenic potential (CNP) contents of the raw roots. Hence, the correlation of the end‐user preferences with the chemical composition of the cassava roots could be helpful to breeders in refining selection criteria and developing high‐throughput screening methods.

## Graphical abstract

Sensory attributes of cassava semolina (gari and eba).

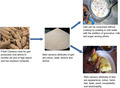

## Introduction

Because of their inherent high moisture content (60–70% wet basis), cassava roots are subject to rapid microbial and physiological deterioration after harvest, leading to undesirable biochemical changes (Onyenwoke & Simonyan, 2014). As a result, value is added to the roots through processing to improve the palatability, increase shelf‐life, facilitate transportation, as well as detoxify the roots by removing cyanogenic compounds (Westby, 2002; Nyirenda *et al*., 2011). In African countries in general and Nigeria, Ghana, Benin, Togo and Côte d'Ivoire, cassava roots are used in a wide range of food products such as *gari*, *tapioca*, *lafun*, *fufu*, starch and *attieke*. All these products differ in their functional, pasting and sensory characteristics, which are mostly influenced by the processing methods used (Sanni *et al*., 2003; Onitilo *et al*., 2007). Processing cassava roots confers a range of specific functional properties to each of the end‐products. Which are determined by various biophysical processes, for instance, starch gelatinisation (Sánchez *et al*., 2010), degradation of cell wall components such as pectin (Eggleston and Asiedu, 1994) and formation of semolina‐like particles or thick paste depending on the type of product.

Gari is a dry, crispy, creamy‐white or yellow, granular flour (semolina) obtained from cassava roots by peeling, washing, grating, pressing, fermenting (optional), sieving and roasting (Escobar *et al*., 2018). It is the most consumed and traded of all cassava food products in Nigeria (FOS, 1970; Westby & Twiddy, 1992; Okafor *et al*., 1998), and in many other countries in West Africa (Oduro *et al*., 2000). The extensive consumption of gari has been attributed to its relatively long shelf‐life compared to other food products from cassava and its ease of preparation before consumption (Omueti *et al*., 1993; Sanni *et al*., 2005).

Gari is usually consumed in the uncooked form, or added with water, sugar, groundnuts and/or cashew nuts, or cooked into a dough called *eba*—the most widely eaten form or sprinkled on cooked cowpea beans in some Africa countries like Nigeria, Togo and Benin Republic (Adinsi *et al*., 2019). Eba is made by sprinkling gari into a bowl or pot of boiled water and continue stirring until dough is formed. Eba is served with vegetable soup and fish or meat. Most households widely purchase gari because of its quick ability to make eba without going through any form of stress (Ogundipe *et al*., 2013).

In many developing countries of the world, gari has become an outstanding staple food for many households. The main consumption areas of gari in Africa are Nigeria, Benin, Togo and Ghana. The other marginal Africa's consumption markets are Niger, Burkina Faso, Ivory Coast, Guinea, Chad, Gabon, Congo and Cameroon (Africabiz online, 2020). In Nigeria, gari processing firms occupy a substantial portion of small and medium enterprises (SMEs) that has contributed significantly to national economic growth (Ogundipe *et al*., 2013). Gari production in Nigeria accounts for about two‐thirds of fresh (unpeeled) cassava roots (Adeoti *et al*., 2009). An average household of eight persons consumed 25 kg of gari at the cost of N2, 500 representing 5% of monthly household income in Ohaozara, Ebonyi State, Southeast Nigeria (Onyemauwa, 2010). Nigerian gari supplies Niger Republic, Chad and Cameroon (Coulibaly *et al*., 2014). Though, the annual gari production in Cameroon is around 49 000 tons, representing about 43 300 million USD (AGROPME, 2010; FAO, 2018). In terms of market volume and value, gari represents 45% of the Cameroonian national market of cassava products, and up to 53% in urban areas, where the demand is significant, with almost 74% of households consuming gari (PNDRT, 2006; Tolly Lolo, 2013). Gari consumption in Cameroon is most strongly associated with people originating from the South‐West and North‐West Regions (Njukwe *et al*., 2013). It may be related both to the geographical proximity of these two regions with Nigeria, which is the largest gari producer and consumer and to common colonial heritage. The extensive consumption of gari has been attributed to its relatively long shelf‐life compared to other food products from cassava and its ease of preparation before consumption (Omueti *et al*., 1993; Sanni *et al*., 2005). The total quantity of fresh cassava roots used for the production of gari in Republic of Benin, Togo and Ghana as at 2003 is 3, 160, 2385 and 9309 metric tons respectively. The total quantity of gari consumed by these countries as at 2003 is Republic of Benin 210.73 metric tons, Togo 159.06 metric tons and Ghana 620.66 metric tons (Africabiz online, 2020). Considering the readiness of gari to be used as diet's complement for a variety of African sauces and cooking and, the long shelf‐life under normal atmospheric conditions, it is possible to expand the consumption area of gari to covering Central, Eastern and Southern African countries. Table 1 showed some of the gari consuming countries.

**Table 1:** Major Gari consuming countries in Africa

S/no.	Countries	Local names	Consumption forms
1	Nigeria	Gari/Garri	Eaten raw and cooked (eba)
2	Liberia	Gari/farina	Eaten as snacks
3	Republic of Benin	Gari	Eaten raw and cooked (eba)
4	Togo	Gari	Eaten raw and cooked (eba)
5	Burkina Faso	Gari	Eaten as snacks
6	Ghana	Gari	Eaten raw and cooked (eba)
7	Mozambique	Rale	Eaten as snacks
8	Cameroon	Gari	Eaten as snacks
9	Ivory Coast	Gari	Eaten raw and cooked (eba)

*Sources*: Abass *et al*.(2012); Coulibaly *et al*.(2014); Awoyale *et al*.(2019); Africabiz online (2020).

Differences in cassava varieties have been reported to play important roles in the production of diversified food products and have significantly affected the physicochemical, functional, sensory and other quality characteristics of gari (Montagnac *et al*., 2009). The knowledge of the suitability of different cassava varieties for gari could contribute to reducing the challenge of how to balance the requirements of farmers with those of processors and end‐users, particularly where there may be a compromise in productivity for varieties with the highest expression of the processor‐ and consumer‐preferred qualities (Egesi *et al*., 2012). Thus, this review describes gari end‐user preferences in the form of uncooked or cooked gari (eba), implications for cassava trait evaluation, and research needed to meet the demands of gari consumers.

## Processing of cassava roots into gari

The details of the processing of cassava roots into gari differ from one location to another, depending on regional preferences, resulting in a large family of different types of gari. Producers/consumers may prefer gari with a sour, sweet or bland taste; a fine or coarse particle size; with or without palm oil added; or even gari enriched/fortified with different legumes or protein sources (Abass *et al*., 2012; Awoyale, 2018; Olaleye *et al*., 2018).

Gari processing involves various steps that include the peeling of fresh cassava roots, washing, grating, fermenting (optional), dewatering/pressing, pulverising, sifting, roasting, sieving or grading and packaging (Abass *et al*., 2012). Manual peeling of freshly harvested roots with a knife is most common, but mechanical peelers are now available in countries such as Nigeria and Ghana (Abass *et al*., 2012). The brown peel, if not removed or partly removed, might adversely affect the gari colour and increase its fibre content. Washing of the peeled roots is done to remove all extraneous materials, which could contaminate the gari. Grating of the washed cassava roots is generally done using a motorised cassava grater. However, hand graters, made by fastening a perforated grating sheet onto a wood slab, are still used in some countries. The resulting product is a wet mash. Grating increases the surface area of the root pieces so that dewatering of the mash can be done more quickly. The grated cassava mash is bagged using a polypropylene/polyethylene woven permeable bag or basket (lined with polypropylene sack) and left for between one and five days to ferment. Fermentation time is based on location: for example, consumers in South‐west Nigeria do prefer sour gari, unlike those in the South‐south and South‐east. Apart from the taste, fermentation helps to reduce the cyanogenic potential of the product (Abass *et al*., 2012). The fermented mash is dewatered by pressing with a manual screw or hydraulic press (often car jacks are used) or even wood pieces tied at both ends with rope, which is still prevalent in most rural communities. Pressing is done to reduce the moisture content of the grated mash before roasting. The cake formed after dewatering is pulverised by a pulveriser/cake breaker or by hand and sieved with a standard woven sieve or rotary sieve, to remove the fibre and lumps, and to create a grit of similar particles size. However, in some locations, after the pulverisation, generally with the grating machine, the grit is not sieved before roasting.

The sieved grit is then roasted. An earthenware stove and a roasting pan made of moulded aluminium or stainless steel are used for roasting on a wood fire (Abass *et al*., 2012). In some communities, the roasting pan is smeared with a small amount of palm oil before roasting, to produce butter‐coloured or yellow gari. Mechanical roasters are also now available in Nigeria and Ghana. The roasting process further develops the gari flavour, gelatinises the starch, and improves digestibility. The extent of drying determines the crispiness and storability of the product. Because starch in the grit is gelatinised during roasting, gari is a pre‐cooked instant food product. In some communities, the grit is partially toasted and finally dried under the sun. Sun drying, while economical, adds risk that the product might be contaminated with dust and sand. The gari is then allowed to cool for some hours, graded (sieved) depending on the particle sizes to meet the preferences of different categories of the consumers and packaged according to the distribution outlet, from wholesale to retail. Most rural communities package in 50 kg bags for transport and distribution. The sensory and functional properties of gari always result from the combination of raw materials (fresh cassava roots) quality and processing operations. The diversity of processing technologies and the resulting diversity of gari have been documented in the scientific literature. On the other hand, the relative influence of cassava roots characteristics and processing on the quality and consumers acceptability of the end‐product remains to be investigated in details.

The challenge with the consistent consumption of gari is its poor nutritional value, which is also common with all cassava products. Gari is known for its high carbohydrate (starch) content, but with low protein, fat and micronutrients contents. The regular consumption of low protein gari can predispose consumers to protein‐energy malnutrition (Alozie & Ekerette, 2017). Consequently, the enrichment of gari with protein‐rich plant foods (soy beans, groundnut, seasame seed and melon seed) has been reported to improve the nutritional quality and sensory acceptability (Arisa *et al*., 2011; Oluwamukomi & Jolayemi, 2012; Oluwamukomi, 2015a, 2015b; Alozie & Ekerette, 2017). Gari enriched with palm oil and/or soybean was developed in Benin Republic, but the gari are not available on the Beninese markets, whereas they are readily available in Nigeria and their processes (Akinoso & Olatunde, 2014), physicochemical characteristics (Edem *et al*., 2001; Karim *et al*., 2016) and sensory properties (Sanni & Sobamiwa, 1994; Osho, 2003) are documented. In most Nigerian enriched gari, the ingredients (palm oil and/or soybean) are usually added to the fermented mash prior to roasting, whereas in Benin Republic, the palm oil/or soybean are added in the mash before fermentation (Adinsi *et al*., 2019). Awoyale *et al*. (2019) also reported that the practice of adding moringa leaf powder, groundnut paste, roasted coconut chips and milk powder to gari in Liberia caused the increase of fat and protein contents of the product compared to the unenriched white gari.

Apart from the artificial enrichment of gari, biofortified cassava varieties that contain significant levels of pro‐vitamin A carotenoids have been developed by conventional plant breeding methods and released for use by the local populations for gari production among other cassava value added products. Gari produced from the biofortified varieties may help solve the issue of the additional cost of adding palm oil and the occurrence of rancidity in the use palm oil while contributing to the reduction of vitamin A deficiency (Bechoff *et al*., 2018). However, understanding how the pro‐vitamin A carotenoids in the biofortified varieties degrade during storage of vitamin A‐containing gari is critical because it will affect its nutritional impact (Bechoff *et al*., 2018). Onadipe (2011) studied the degradation of total carotenoids in gari from different biofortified cassava varieties and found out that 50% on average of total carotenoids were lost after 3‐month storage at 30 ± 2 °C. Eyinla *et al*. (2019) on their own part reported that processing biofortified cassava into gari and to eba could hinder the retention of β‐carotene, though some varieties have retention advantage over others irrespective of the initial concentration in the fresh roots.

## Gender implications for variety development for gari/eba quality

Women play a central role in cassava production in most African countries. In Nigeria, harvesting, processing and marketing, are contributing about 58% of the total agricultural labour in the Southwest, 67% in the Southeast and 58% in the central zones (FAO, 2004; Onyemauwa, 2012). After gari processing in Nigeria, women sell a kongo (Nigeria government‐approved measurement of 1.2 kg) at the rate of N100 and a plastic bowl of gari (8–9 Kongo) at the rate of N600. The women also give out gari to relations in urban areas for free (Butterworth *et al*., 2008). Some processing groups also employ women, and some youth to help fetch water. The wages for the activities range from N300 to N600 per day, excluding meals (Butterworth *et al*., 2008). Women rely mostly on traditional techniques to produce gari in Cameroon. Cassava transformation to gari through the use of modern technology could spur rural development, and hence, raise incomes for producers, processors and traders, and consequently contribute to national food security in Cameroon (Lengha, 2017). About 84% of those involved in gari production in Bamunkumbit, Northwest Cameroon are women (Lengha, 2017). In Liberia, the processing of cassava roots into gari is mainly traditional and largely dominated by women. Industrialisation of gari processing is limited and would require investments in hardware, training and promotion. Women of relatively young age direct about 60% of the gari processing enterprises in Liberia. These women have short experience in gari production. About 60% of the women involved in gari production belong to an organisation. The main advantages of belonging to this organisation are access to credit, input and information among others (Coulibaly *et al*., 2014). In Sierra‐Leone, a significant number of farmers who are women process cassava roots into gari and other minor products such as fufu and kondogbala, using locally made grating and the pressing machines. Processing of cassava roots into gari is done traditionally at the farm‐gate, community and processor levels in Sierra‐Leone (Coulibaly *et al*., 2014). It is the alcohol and gari production which provide the most important economic benefit in Sierra‐Leone (Coulibaly *et al*., 2014). Gari production, which serves to address the problem of postharvest losses and generating income, is an important source of livelihood for many women between the age of 30 and 75 years in the informal sector in Ghana (Nimoh *et al*., 2020). Nimoh *et al*. (2020) also reported that gari production in Kumasi Ghana is financially profitable, with all the profitability indicators showing positive returns on inputs employed in production. However, the constraints identified in the gari production business in Ghana are seasonality and high cost of cassava roots, which may be addressed by cost‐effective management strategies and release of all year round cassava varieties (Nimoh *et al*., 2020). The involvements of women in the production of gari in other African countries like Benin, Togo and Côte d’Ivoire among others need proper documentation.

## Sensory analysis and consumer preference of gari

Sensory criteria constitute a significant determinant of acceptability of any cassava variety, along with agronomic characteristics, and have a significant impact on the subsequent adoption and use of a variety for gari production (Idowu & Akindele, 1994; Oyeyinka *et al*., 2019). Both varietal effects and differences in the processing of gari contribute to variants in the sensory attributes of the final product to meet local preferences and traditions (Bechoff *et al*., 2018). Noticeable variability is observed among traditional gari types, depending on processes used, which can confer different sensory properties (colour, particle size, dryness and sourness). This inconsistency in gari quality, documented in Nigeria and Ghana (Oduro *et al*., 2000; Makanjuola *et al*., 2012), is partly because the fermentation process is now often reduced from the traditional four days to just one day to save time and ensure quick returns. Lesser time of fermentation results typically in a less sour gari and the degree of sourness desired depends on location. Speeding up fermentation can be achieved by additives or increased temperature. However, the vast bulk of gari is processed by smallholders who rarely use such technology. We identified several studies that inventoried and described the most relevant sensory attributes to describe consumers' perceptions of gari, eba and enriched gari.

### Sensory acceptability of dry (uncooked) gari

Gari is consumed in the uncooked form by either soaking in water with the addition of milk, sugar, groundnuts and/or cashew nuts in Nigeria, Ghana, Benin and Togo (Udofia *et al*., 2011) or mixed with groundnut paste or moringa leaf powder and consumed as snacks in Liberia (Awoyale *et al*., 2019). The sensory attributes of the gari are affected by many factors. Processing‐related factors include the level of fermentation, roasting temperature, the quantity of palm oil added, postharvest storage of cassava roots before processing, method of grating, and rate of dewatering of cassava mash during fermentation, and storage condition of gari before consumption. Agronomic‐related factors include soil quality, weather conditions, age of the cassava plant at harvest, cassava variety (Collard & Levi, 1959; Okafor & Uzuegbu, 1987; Oduro et al., 2000; Udofia *et al*., 2011). For instance, Adinsi *et al*. (2019a) reported that a positive correlation exists between roasting time and the sensory texture of the raw gari during chewing (*r* = 0.99), as well as between sour taste and fermented odour (0.96). In Nigeria, gari quality varies along traditional/cultural lines. Yellow (addition of palm oil) and sweet (short fermentation time) gari is generally preferred in the East.

Nonetheless, Owuamanam *et al*. (2011) reported that those consumers generally accepted gari fermented for 48 h in the Eastern part of Nigeria who prefers a fermented gari. In the West, creamy to slightly golden coloured and sour gari, imparted by a more extended period of fermentation, is preferred (Udofia *et al*., 2011). This difference may be related to the better storability of white gari, which does not contain oil, and is thus less susceptible to oxidation. On the contrary, yellow gari can readily oxidise during storage.

The primary sensory attributes of dry gari are appearance, colour, taste, acidity, sweetness, flavour/aroma and crispiness (Table 2; Owuamanam *et al*., 2011; Apea‐Bah *et al*., 2011; Makanjuola *et al*., 2012; Udoro *et al*., 2014; Laya *et al*., 2018). Consumer acceptability of gari samples collected from different processing centres across South‐west Nigeria showed that preferences were generally based on colour (*r* = 0.98), taste (*r* = 0.96) and flavour (*r* = 0.99; *P* < 0.01; Makanjuola *et al*., 2012; Table 3).

**Table 2:** Sensory attributes of different types of *gari* and cooked paste (*eba*).

Samples	Appearance	Colour	Texture	Taste	Acidity	Sweetness	Flavor/aroma	Mouldability	Stretchability	Crispiness/mouthfeel	Acceptance	References
*Gari* evaluated in dry form												
Nwanyi bekee local variety (0 h fermented)	5.80	–	–	5.70	–	–	–	–	–	–	5.70	Owuamanam *et al*. (2011)^†^
Nwanyi bekee local variety (12 h fermented)	5.70	–	–	5.70	–	–	–	–	–	–	5.60	Owuamanam *et al*. (2011)^†^
Nwanyi bekee local variety (24 h fermented)	6.30	–	–	6.20	–	–	–	–	–	–	5.50	Owuamanam *et al*. (2011)^†^
Nwanyi bekee local variety (36 h fermented)	6.00	–	–	6.00	–	–	–	–	–	–	5.50	Owuamanam *et al*. (2011)^†^
Nwanyi bekee local variety (48 h fermented)	5.60	–	–	5.60	–	–	–	–	–	–	5.70	Owuamanam *et al*. (2011)^†^
Nwanyi bekee local variety (72 h fermented)	5.20	–	–	5.00	–	–	–	–	–	–	5.10	Owuamanam *et al*. (2011)^†^
Processing centre 1	–	5.43	–	4.57	–	–	5.17	–	–	–	5.40	Makanjuola *et al*. (2012)^†^
Processing centre 2	–	6.50	–	5.83	–	–	5.67	–	–	–	5.93	Makanjuola *et al*. (2012)^†^
Processing centre 3	–	4.90	–	5.03	–	–	4.83	–	–	–	4.80	Makanjuola *et al*. (2012)^†^
Processing centre 4	–	6.45	–	6.37	–	–	6.27	–	–	–	6.23	Makanjuola *et al*. (2012)^†^
Processing centre 5	–	5.37	–	5.08	–	–	5.17	–	–	–	5.07	Makanjuola *et al*. (2012)^†^
Processing centre 6	–	7.13	–	7.13	–	–	7.18	–	–	–	7.38	Makanjuola *et al*. (2012)^‡^
Processing centre 7	–	6.00	–	5.72	–	–	5.50	–	–	–	5.92	Makanjuola *et al*. (2012)^‡^
Processing centre 8	–	8.00	–	7.93	–	–	7.70	–	–	–	8.10	Makanjuola *et al*. (2012)^‡^
Bitter cassava	–	6.85	–	6.45	–	–	6.60	–	–	6.50	6.70	Udoro *et al*. (2014)^†^
TMS92/0326 (12 months)	–	3.45	–	–	2.80	2.55	3.40	–	–	2.55	3.75	Laya *et al*. (2018)*
TMS96/1414 (12 months)	–	2.85	–	–	2.65	2.65	3.20	–	–	2.95	3.80	Laya *et al*. (2018)*
IRAD4115 (12 months)	–	2.75	–	–	2.20	2.50	3.05	–	–	3.30	2.75	Laya *et al*. (2018)*
EN local variety (12 months)	–	4.50	–	–	1.60	2.60	3.50	–	–	2.75	4.30	Laya *et al*. (2018)*
AD local variety (12 months)	–	3.55	–	–	1.65	2.30	2.95	–	–	1.90	3.10	Laya *et al*. (2018)*
TMS92/0326 (15 months)	–	2.50	–	–	1.90	2.30	1.80	–	–	4.30	2.55	Laya *et al*. (2018)*
TMS96/1414 (15 months)	–	2.15	–	–	2.00	2.25	2.50	–	–	3.25	2.75	Laya *et al*. (2018)*
IRAD4115 (15 months)	–	2.40	–	–	1.85	3.00	2.35	–	–	3.15	2.40	Laya *et al*. (2018)*
EN local variety (15 months)	–	2.20	–	–	1.95	3.35	2.55	–	–	3.60	2.50	Laya *et al*. (2018)*
AD local variety (15 months)	–	3.40	–	–	1.65	2.05	2.95	–	–	1.85	3.00	Laya *et al*. (2018)*
Commercial *gari* dry form	–	3.90	–	2.50	2.50	2.95	3.15	–	–	2.85	3.55	Laya *et al*. (2018)*
*Gari* evaluated in uncooked and cooked form (*eba*)												
Bitter cassava	–	6.80	6.50	6.55	–	–	6.55	6.60	–	–	6.60	Udoro *et al*. (2014)^†^
TMS 30572 (0 week storage)	–	5.09	–	4.73	–	–	5.27	–	5.27	–	5.18	Eje *et al*. (2015)^†^
TMS 50395 (0 week storage)	–	5.27	–	5.37	–	–	4.82	–	5.09	–	5.00	Eje *et al*. (2015)^†^
TMS 4(2)1425 (0 week storage)	–	5.18	–	5.73	–	–	5.27	–	4.91	–	5.18	Eje *et al*. (2015)^†^
TMS 91934 (0 week storage)	–	5.00	–	4.64	–	–	5.00	–	5.00	–	5.00	Eje *et al*. (2015)^†^
TMS 30572 (3 week storage)	–	5.18	–	4.27	–	–	4.55	–	4.73	–	4.09	Eje *et al*. (2015)^†^
TMS 50395 (3 week storage)	–	4.45	–	4.55	–	–	4.18	–	4.18	–	4.00	Eje *et al*. (2015)^†^
TMS 4(2)1425 (3 week storage)	–	5.09	–	5.64	–	–	6.00	–	4.36	–	4.55	Eje *et al*. (2015)^†^
TMS 91934 (3 week storage)	–	4.27	–	4.45	–	–	4.73	–	4.64	–	4.64	Eje *et al*. (2015)^†^
TMS 30572 (6 week storage)	–	5.36	–	4.27	–	–	5.18	–	4.27	–	5.00	Eje *et al*. (2015)^†^
TMS 50395 (6 week storage)	–	5.09	–	5.00	–	–	5.09	–	4.73	–	5.00	Eje *et al*. (2015)^†^
TMS 4(2)1425 (6 week storage)	–	4.82	–	4.64	–	–	5.64	–	4.18	–	4.55	Eje *et al*. (2015)^†^
TMS 91934 (6 week storage)	–	5.18	–	4.55	–	–	4.73	–	5.00	–	5.00	Eje *et al*. (2015)^†^
TMS 30572 (9 week storage)	–	4.64	–	4.00	–	–	4.36	–	4.18	–	4.73	Eje *et al*. (2015)^†^
TMS 50395 (9 week storage)	–	5.00	–	4.36	–	–	4.00	–	4.45	–	4.00	Eje *et al*. (2015)^†^
TMS 4(2)1425 (9 week storage)	–	5.00	–	5.27	–	–	5.27	–	4.00	–	4.82	Eje *et al*. (2015)^†^
TMS 91934 (9 week storage)	–	4.55	–	4.45	–	–	4.00	–	4.82	–	4.36	Eje *et al*. (2015)^†^
TMS 30572 (12 week storage)	–	4.91	–	3.86	–	–	5.00	–	5.00	–	4.70	Eje *et al*. (2015)^†^
TMS 50395 (12 week storage)	–	4.64	–	4.55	–	–	4.55	–	5.36	–	3.96	Eje *et al*. (2015)^†^
TMS 4(2)1425 (12 week storage)	–	4.80	–	3.55	–	–	4.18	–	3.27	–	3.96	Eje *et al*. (2015)^†^
TMS 91934 (12 week storage)	–	4.70	–	3.82	–	–	2.73	–	5.40	–	2.45	Eje *et al*. (2015)^†^
TMS 30572 (15 week storage)	–	1.91	–	3.85	–	–	3.18	–	4.00	–	1.18	Eje *et al*. (2015)^†^
TMS 50395 (15 week storage)	–	3.55	–	3.80	–	–	3.95	–	5.18	–	3.45	Eje *et al*. (2015)^†^
TMS 4(2)1425 (15 week storage)	–	1.27	–	2.18	–	–	2.45	–	3.27	–	2.09	Eje *et al*. (2015)^†^
TMS 91934 (15 week storage)	–	2.18	–	2.73	–	–	2.55	–	5.36	–	2.36	Eje *et al*. (2015)^†^
100% cassava	–	7.23	6.56	7.13	–	–	7.02	–	–	–	7.43	Oluwamukomi (2015a, 2015b)^‡^
5% Full fat sesame	–	4.30	3.55	3.00	–	–	4.05	–	–	–	4.30	Oluwamukomi (2015a, 2015b)^‡^
10% Full fat sesame	–	3.00	3.80	3.50	–	–	4.40	–	–	–	3.60	Oluwamukomi (2015a, 2015b)^‡^
5% defatted sesame	–	3.90	4.95	3.90	–	–	5.05	–	–	–	4.70	Oluwamukomi (2015a, 2015b)^‡^
10% defatted sesame	–	5.30	5.10	5.35	–	–	5.80	–	–	–	5.35	Oluwamukomi (2015a, 2015b)^‡^
Bitter cassava	5.73	6.00	6.20	5.73	–	–	5.53	–	–	–	6.20	Olaleye *et al*. (2018)^‡^
Sweet cassava	6.47	6.07	6.53	7.00	–	–	6.53	–	–	–	7.20	Olaleye *et al*. (2018)^‡^

Texture: the texture of the dried *gari* is the crispiness, while that of the *eba* is the hand feel before consumption.

Taste: This is the combination of sweetness or sourness of the *gari*.

* 5‐point hedonic scale.

^†^ 7‐point hedonic scale.

^‡^ 9‐point hedonic scale.

**Table 3:** Pearson correlation of sensory attributes and consumers acceptability

Sensory evaluations	Colour	Taste	Acidity	Sweetness	Flavour/odour	Texture	Appearance	Crispiness/mouthfeel	Stretchability	Acceptability
Effect of length of fermentation on sensory acceptability of gari (Olaoye *et al*., 2015)										
Appearance	–	0.98**	–	–	–	–	1.00	–	–	0.47
Taste	–	1.00	–	–	–	–	0.98**	–	–	0.57
Acceptance	–	0.57	–	–	–	–	0.47	–	–	1.00
Effect of varieties and period of harvest on sensory acceptability of soaked gari (Laya *et al*., 2018)										
Colour	1.00	–	–0.19	–0.31	0.74*	–	–	–0.63*	–	0.84**
Acidity	–0.9	–	1.00	0.15	0.30	–	–	0.14	–	0.27
Sweetness	–0.31	–	0.15	1.00	–0.06	–	–	0.38	–	–0.21
Odour	0.74*	–	0.30	–0.07	1.00	–	–	–0.66*	–	0.84**
Acceptance	0.81**	–	0.27	–0.16	0.83**	–	–	–0.44	–	1.00
Effect of different processing centres on the sensory acceptability of gari (Makanjuola *et al*., 2012)										
Colour	1.00	0.96**	–	–	0.97**	–	–	–	–	0.98**
Taste	0.96**	1.00	–	–	0.97**	–	–	–	–	0.96**
Flavor	0.97**	0.97**	–	–	1.00	–	–	–	–	0.99**
Acceptance	0.98**	0.96**	–	–	0.99**	–	–	–	–	1.00
Effect of cassava varieties and storage on sensory acceptability of eba (Eje *et al*., 2015)										
Colour	1.00	0.75**	–	–	0.77**	–	–	–	0.27	0.85**
Taste	0.75**	1.00	–	–	0.82**	–	–	–	0.25	0.71**
Aroma	0.77**	0.82**	–	–	1.00	–	–	–	0.07	0.87**
Stretchability	0.27	0.25	–	–	0.07	–	–	–	1.00	0.19
Acceptance	0.85**	0.71**	–	–	0.87**	–	–	–	0.19	1.00
Effect of sesame enrichment on the sensory acceptability of eba (Oluwamukomi, 2015a, 2015b)										
Colour	1.00	0.92*	–	–	0.90*	0.87	–	–	–	0.98**
Texture	0.87	0.95*	–	–	0.98**	1.00	–	–	–	0.94*
Taste	0.92*	1.00	–	–	0.99**	0.95*	–	–	–	0.95*
Flavor	0.90*	0.99**	–	–	1.00	0.98**	–	–	–	0.95*
Acceptance	0.98**	0.95*	–	–	0.95*	0.94*	–	–	–	1.00

–, not evaluated.

The sample size used for the Pearson correlation is the mean of each parameters presented by the authors.

* *P* < 0.05.

** *P* < 0.01.

One study (Laya *et al*., 2018) found that consumer acceptability of uncooked gari after soaking in sugary water (10% sucrose) was correlated mainly with colour and odour (*r* = 0.83, *P* < 0.01) (Table 3). Additionally, the age of the roots appeared to play a role, as consumers preferred gari from cassava roots harvested at 12 months after planting to that from roots harvested at 15 months.

### Sensory acceptability of cooked gari (eba)

Gari can be rehydrated by adding boiling water and made into dough called eba. Eba is the most consumed cassava product in Nigeria and some other West Africa countries. Eba is made by sprinkling raw gari into a bowl or pot of boiled water and continue stirring until dough is formed. It is served with a vegetable or preferred soup (Ogundipe *et al*., 2013). The primary sensory attributes of eba are appearance, colour, texture, taste, aroma, mouldability and stretchability as shown in Table 2 (Udoro *et al*., 2014; Eje *et al*., 2015; 2015a, 2015b; Olaleye *et al*., 2018). The factors that affect the sensory acceptability of uncooked gari also affect that of eba, in addition to the quantity of boiled water used for reconstitution and the uniformity in the stirring process. For instance, Eje *et al*. (2015) studied the sensory acceptability of gari and eba produced from different improved varieties of fresh cassava roots, either stored in layers in moist sawdust of 80% moisture content (wb), or unstored. They observed that there were no significant differences (*P* > 0.05) in the stretchability of the eba from any of the varieties either stored or unstored. The result was attributed to un‐degraded starch in the cassava root stored in the moist sawdust. Also, gari produced from the unstored cassava varieties were most acceptable in terms of colour (*r* = 0.85), taste (0.71) and aroma (*r* = 0.87; Table 3).

### Enriching gari to improve sensory acceptability

The enrichment of gari with defatted and full‐fat sesame seed flour at different ratios showed that 10% defatted sesame seed flour‐enriched gari cooked into eba compared favourably with the control sample (no enrichment; Oluwamukomi, 2015a, 2015b). The acceptability of this type of eba sample may be attributed to the colour, texture, taste and flavour. There was a significant positive correlation (*r* = 0.90, *P* < 0.05) exists between consumer acceptability and the sensory attributes (Table 3). Adinsi *et al*. (2019) observed that the addition of soybean and/or palm oil did not affect the appearance, odour, texture and taste of the traditional raw gari. Sanni *et al*. (2010) reported no significant difference (*P* > 0.05) in the taste, texture and odour of iron‐fortified vs unfortified gari. Karim *et al*. (2016) reported that gari produced from the grating of 90% fresh cassava roots and 10% fresh sweet potato roots were most preferred in the overall acceptability compared to 100% fresh cassava gari. Also, the mouldability and overall acceptability of eba made from gari samples of 90% fresh cassava root and 10% sweet potato roots were higher compared to that of the 100% fresh cassava root gari (Karim *et al*., 2016). Roasted soy gari was reported by Ogunlakin *et al*. (2015) to be more preferred for both the raw (uncooked) and cooked form (eba) compared to raw gari and eba from 100% cassava roots.

## Processing methods, product characterisation and relationship with sensory evaluation

### Root composition and processing methods related to product quality

While the characteristics of the raw material (cassava roots) are essential, processing also plays a crucial role in determining the quality of gari and its sensorial perception by consumers. In particular, the grating and roasting operations determine particle size and hence the structure and texture of the final product. In contrast, the fermentation operation changes the pH and composition in organic acids and therefore the taste of the final product. The scientific literature reports the following key findings on how processing influences gari quality.

Gari processing leads to high nutrient losses due to leaching out with water, and roasting due to thermal degradation. Favier *et al*. (1969) estimated these losses at around 22% for carbohydrates and above 50% for several other nutrients and micronutrients. Nutrient losses may vary depending on the type of technology used, from completely traditional and manual to semi‐mechanised. Aloys & Zhou (2005) indicated that longer fermentation of raw cassava is associated with several traits of the processed gari: higher yield, higher density, higher dispersibility, higher crude fibre, and higher pasting temperature, but lower pH, starch, cyanide content, peak viscosity, paste viscosity and water retention of the gari. About 80% of the dry matter in cassava root consists of carbohydrates, including starch in the majority, fibre and sugars (Kim *et al*., 1995; Huang *et al*., 2007; Goddard *et al*., 2015). The starch itself consists of amylose (a linear‐chain polymer of glucose units) and amylopectin (branched‐chain of glucose units).

In native starch, amylose and amylopectin are arranged in semi‐crystalline granules. The role of starch in determining the texture of cassava‐based products is debated in the scientific literature and appears to depend on the product considered. For starch suspensions in excess water, the increase in viscosity during cooking and subsequent cooling is correlated with the shape and size of starch granules, swelling power and the amylose‐to‐amylopectin ratio (Sánchez *et al*., 2010). For semi‐solid systems composed of pure starch, that is starch gels, the texture and rheological properties were influenced by the branched structure of amylopectin (Charles *et al*., 2005). On the contrary, in more complex systems such as boiled roots, the molecular structure of starch was not related to textural properties (Charoenkul *et al*.,^2006^), which suggested that other components (fibres, cell wall materials, etc.) may play a more critical role (Favaro *et al*., 2008).

In the case of gari, starch appears to play a significant role in determining the texture of the end‐product (gari as well as eba). During the roasting operation, starch gelatinisation results in swelling and eventual break down of starch granules upon absorption of water to form an amorphous matrix. Starch properties such as water absorption capacity after gelatinisation and the amylose: amylopectin ratio underpin the rheological and textural behaviour of the starch matrix, and hence contribute to explain the textural characteristics of gari, as well as the pasting properties of eba (Akingbala *et al*., 2005; Goddard *et al*., 2015). Besides, Maieves *et al*. (2012) reported that cassava varieties whose starch granules are more bound with parenchyma tissues, pectin and cellulose tend to be harder in texture, both in raw and in cooked cassava roots, which may also affect the quality of the gari and eba. Thus quantification of starch and fibre could help to predict the suitability of cassava roots for production of gari, especially when considering that the age of the plants can influence the starch and fibre contents of the product. Thus, cassava of not more than 12 months should be used for the production of gari because of the high starch content and low fibre content varies between cassava varieties and with the age of the plants. The viscosity of cassava‐based products has been correlated with starch granule shape and sizes, swelling power and the amylose and amylopectin ratios (Sánchez *et al*., 2010).

On the contrary, Charoenkul *et al*. (2006) stated that textural properties were not related to the molecular structure of starch. This was also supported by the observation of Charles *et al*. (2005), based on a visual assessment of texture rather than on instrumental methods. Also, a positive correlation between particle size and moisture content of gari was reported by Makanjuola *et al*. (2012). This implied that gari with large particle sizes would be associated with higher moisture content and thus, with a problem of storage stability. Aryee *et al*. (2006) stated that cassava varieties of poor cooking quality and high cyanogenic potential could be used for the production of starch, glucose, adhesives, fuel, alcohol and other industrial materials, but not primary cassava product like gari. Gouado *et al*. (2008) showed that increasing frying time above 10 min, reduces the acceptability of gari. Frying temperature, the quantity of palm oil added, storage conditions of cassava roots, grating method and dewatering conditions of cassava mash are other technological factors influencing the acceptability of gari (Oduro *et al*., 2000; Udofia *et al*., 2011).

Another set of publications on gari processing in the scientific literature deal with the nutritional enrichment (fortification) of gari, and how to minimise losses in nutritional value during processing. National and international research centres such as the International Institute of Tropical Agriculture (IITA) and the National Root Crop Research Institute (NRCRI) in Nigeria have developed biofortification programmes (e.g. HarvestPlus) to increase vitamin A, iron and zinc in basic food crops such as cassava, maize, beans and potatoes to reduce micronutrient deficiency in Sub‐Sahara Africa (IITA, 2011). The consumer acceptability of some traditional food products from biofortified crops as affected by different processing methods, especially cassava (e.g. gari and fufu) have been assessed (Omodamiro *et al*., 2012; IITA, 2011; Aniedu & Omodamiro, 2012). Increasing the carotenoids content of cassava through conventional breeding has resulted in yellow‐fleshed varieties with up to 10 µg g^−1^ (wb) total carotenoids, that is two to five times higher than traditional varieties. During postharvest processing, part of the carotenoids is lost due to leaching and molecular degradation. In the case of gari processing, the retention rate was relatively high when evaluated on a wet basis. According to Omodamiro *et al*. (2012), grating yellow‐fleshed cassava root retained 97.7–98.5% of the original total carotenoid content of between 6.3 and 7.8 μg g^−1^. Subsequent fermentation of this mash leads to 94.7–96.7% retention of carotenoids. In another study of improved varieties TMS 01/1371, TMS 01/1235 and TMS 94/0006 into gari, fermentation significantly increased the average carotenoid content from 4.9 to 8.6 μg g^−1^ (wb; Maziya‐Dixon *et al*., 2015). The increase in the proportion of carotenoids content was explained by a reduction in moisture as well as hydrolysis of carbohydrates and fibre by hydrolysis during fermentation. However, Ortiz *et al*. (2011) indicated that dry basis measurements of the carotenoid content would give a more accurate trend of what transpired during fermentation of the cassava mash. Regarding other micronutrients, Maziya‐Dixon *et al*. (2015) also noted that fermentation significantly reduced the iron content from 7.47 to 7.13 mg kg^−1^, and the zinc content from 8.95 to 5.58 mg kg^−1^ (wb). This can be interpreted as the leaching of minerals due to the acidic nature of the fermentate, and oxidative activities of microbes that use the micronutrients for development and growth (Ayetigbo *et al*., 2018). Production of gari from yellow‐fleshed cassava varieties has been reported to retain the least β‐carotene viz: oven‐dried chips (71.9% retention) > shade‐dried chips (59.2%) > boiled roots (55.7%) > sun dried chips (37.9%) > gari production (34.1%; Chavez *et al*., 2007).

### Texture assessment and relationship with sensory evaluation

Understanding the textural properties of foods allows for better control of food operations such as cooking, heating, roasting and drying, attaining the desired quality attributes of the product (Chen & Opara, 2013). For gari and eba, several parameters such as particle size, water absorption capacity and starch properties contribute to texture, resulting in wide variations between gari‐producing regions. The scientific literature provides some information on texture evaluations of gari; however, further work may be useful in order to elucidate the underlying molecular mechanisms. The texture is an essential quality parameter when it comes to the reconstitution of the gari to eba. The most crucial factor influencing cassava product texture is the quantity and quality of starch (Charles *et al*., 2005; Charoenkul *et al*., 2006). Texture assessment of food can be done using either human senses (sensory texture profile analysis‐‐STPA) or instruments (instrumental texture profile analysis‐‐ITPA). For the STPA, consumers are asked to rate the textural attributes resulting from different varieties and of different planting periods, allowing the researcher to identify consumer‐preferred textural attributes and to select varieties with consumer‐preferred characteristics (Tomlins *et al*., 2013), while the ITPA uses equipment designed to imitate the chewing process, providing standardised data through which a wide range of food texture properties can be analysed, including hardness, springiness, adhesiveness, resiliency, fracturability, wateriness, gumminess, sliminess and chewiness (Chen & Opara, 2013; Goddard *et al*., 2015). This implies that the ITPA can significantly expedite the testing process and allow for repeat testing of the same sample over time, and this seems to be the best method for rapid and repeatable assessment of texture. Maieves *et al*. (2012) observed that the use of a texturometer to determine the hardness of cooked cassava roots could significantly facilitate the decision on which varieties are softer for industrial processes involving heat treatment of raw materials. There is a paucity of research on the ITPA of gari produced from different cassava varieties reconstituted to eba, though work has been done on the texture analysis of different cassava products (Asaoka *et al*., 1992; Defloor *et al*., 1998; Tomlins *et al*., 2007; Aryee *et al*., 2006; Anggraini *et al*., 2009; Sánchez *et al*., 2010; Oyewole & Afolami, 2001; Franck *et al*., 2011; Makanjuola *et al*., 2012; Perez *et al*., 1998).

### Relationship between composition and sensory evaluation

The functional properties indicate how the food materials under examination interact with other food components directly or indirectly affecting the processing applications, food quality, and ultimate acceptance by the consumers (Adeleke & Odedeji, 2010). From the work of Awoyale *et al*. (2020), it was observed that a significant positive correlation (*P* < 0.01; *r* = 0.56) exist between the water absorption capacity (WAC) of polypropylene woven sack stored gari samples and the overall acceptability of the eba. Also, the mouldability (*P* < 0.05; *r* = 0.51) and mouthfeel (*P* < 0.01; *r* = 0.46) of the eba were positively correlated with the WAC. The solubility index of gari packaged in polypropylene woven sack had a significant positive correlation (*P* < 0.05, *r* = 0.61) with the overall acceptability of the eba, and a negative (*P* < 0.05) correlation with the peak time (*r* = −0.62) and pasting temperature (*r* = −0.63; Awoyale *et al*., 2020). The bulk density of the gari packaged in polypropylene woven sack was positively correlated (*P* < 0.05, *r* = 0.58) with the mouldability of the eba. The setback viscosity of the gari packaged in polyvinyl chloride container was negatively correlated (*P* < 0.05, *r* = −0.58) with the texture of the eba (Awoyale *et al*., 2020). Furthermore, a study on the effect of varieties and period of harvesting (Laya *et al*., 2018) on the functional properties and sensory attributes of gari revealed that a significant positive correlation (*P* < 0.01) exist between overall acceptability and the bulk density (r = 0.86), water absorption capacity (*r* = 0.83), and swelling power (*r* = 0.77; Table 4). Also, the correlation between the bulk density (*r* = 0.82), water absorption capacity (*r* = 0.67) and the colour of the gari was positive and significant (*P* < 0.05). But, the least gelation concentration of the gari had a negative and significant (*P* < 0.05) correlation with the colour (*r* = −0.66) and the odour (*r* = −0.64; Table 4).

**Table 4:** Pearson correlation of functional properties and sensory evaluation of *gari* from Laya *et al*. (2018) data

Parameters	BD	WAC	SWP	LGC	Colour	Odour	Mouthfeel	Acidity	Sweetness	Acceptance
BD	1.00									
WAC	0.73*	1.00								
SWP	0.68*	0.95**	1.00							
LGC	−0.74*	−0.54	−0.64*	1.00						
Colour	0.82**	0.67*	0.62	−0.66*	1.00					
Odour	0.85**	0.59	0.59	−0.64*	0.74*	1.00				
Mouthfeel	−0.51	−0.25	−0.14	0.34	−0.63*	−0.66*	1.00			
Acidity	0.03	0.31	0.31	0.05	−0.19	0.30	0.14	1.00		
Sweetness	0.05	−0.18	−0.13	0.22	−0.31	−0.06	0.38	0.15	1.00	
Acceptance	0.86**	0.83**	0.77**	−0.54	0.81**	0.83**	−0.44	0.27	−0.16	1.00

BD, bulk density; LGC, least gelation concentration; SWP, swelling power; WAC, water absorption capacity.

* *P* < 0.05.

** *P* < 0.01.

In addition to the functional properties, biochemical composition contributes to the sensory perception and acceptability of gari and eba. Key biochemical factors include the production of lactic acid and other organic acids during fermentation that gives the end‐product its sour taste and the presence of sugars and proteins that contribute to browning during roasting. The acceptability of gari by consumers varies with regional preferences; nevertheless, some correlations can be observed between chemical composition and the acceptability of sensory attributes of gari (Table 5). Laya *et al*. (2018) studied the effect of varieties and the period of the harvest of cassava roots on the sensory evaluation of gari. They found that the acceptability of gari produced from different varieties had a negative but not significant relationship with moisture content (*r* = −0.47), and positive relationship with the lipid (*r* = 0.40, *P* > 0.05) and the carbohydrate (*r* = 0.67, *P* < 0.05) contents (Table 5). In addition, the moisture content of the gari had a negative correlation with the colour (*r* = −0.26, *P* > 0.05), odour (*r* = −0.47, *P* > 0.01),) and mouthfeel (*r* = −0.01, *P* > 0.01), but a positive correlation with sweetness (*r* = 0.13, *P* > 0.01). The protein content was significant and positive only with the odour (*r* = 0.65, *P* < 0.05; Table 5).

**Table 5:** Pearson correlation of chemical composition and sensory evaluation of *gari*

	Moisture	Ash	Protein	Lipid	Crude fibre	CHO	Sugar	CNP	pH
Effect of varieties and period of harvest on sensory acceptability of gari (Laya *et al*., 2018)									
Colour	−0.26	–	0.58	0.10	0.06	0.56	0.24	−0.07	−0.56
Odour	−0.47		0.65*	0.44	−0.34	0.69*	0.40	−0.37	−0.76*
Acidity	−0.38	–	−0.04	0.60	−0.26	0.34	0.71*	−0.82**	−0.54
Sweetness	0.13	–	0.21	−0.24	−0.08	0.22	−0.11	−0.37	0.16
Mouthfeel	−0.01	–	−0.43	−0.22	0.28	−0.05	−0.29	−0.06	0.50
Acceptance	−0.47	–	0.54	0.40	0.01	0.67*	0.38	−0.50	−0.67*
Effect of different processing centres on the sensory acceptability of gari (Makanjuola *et al*., 2012)									
Colour	0.28	−0.13	NA	−0.67	−0.37	NA		NA	NA
Taste	0.06	−0.24	NA	−0.51	−0.19	NA		NA	NA
Aroma	0.12	−0.05	NA	−0.54	−0.40	NA		NA	NA
Acceptance	0.19	−0.04	NA	−0.61	−0.40	NA		NA	NA
Effect of Sesame enrichment on the sensory acceptability of eba (Oluwamukomi, 2015a, 2015b)									
Colour	0.57	−0.31	−0.47	−0.86	0.80	0.81		0.78	−0.52
Texture	0.47	0.00	−0.34	−0.89*	0.81	0.67		0.98**	−0.73
Taste	0.40	−0.18	−0.35	−0.79	0.92*	0.69		0.92*	−0.54
Aroma	0.40	−0.08	−0.31	−0.83	0.88*	0.66		0.96**	−0.60
Acceptance	0.61	−0.23	−0.50	−0.91*	0.83	0.83		0.87	−0.66
Effect of varieties and length of fermentation on sensory acceptability of gari (Olaoye *et al*., 2015)									
Appearance	−0.93**	0.91**	0.60*	−0.76**	0.83**	0.91**		−0.54	NA
Texture	−0.93**	0.93**	0.58	−0.77**	0.79**	0.93**		−0.55	NA
Taste	−0.94**	0.93**	0.55	−0.77**	0.79**	0.95**		−0.56	NA
Aroma	−0.91**	0.95**	0.45	−0.69*	0.78**	0.95**		−0.71**	NA
Acceptance	−0.67*	0.59*	0.33	−0.46	0.59*	0.69*		−0.58	NA

CHO, carbohydrate; CNP, cyanogenic potential; NA, not available.

The Pearson correlation was done using the primary data from the authors by means of SPSS version 21.

* *P* < 0.05.

** *P* < 0.01.

The carbohydrate content of gari has significant positive correlations with the odour (*r* = 0.69, *P* < 0.05) and the overall acceptability (*r* = 0.69, *P* < 0.05). The cyanogenic potential was negatively correlated with the acidity (= −0.82, *P* < 0.01). However, all the sensory attributes have negative but not significant (*P* > 0.05) correlation with the CNP. The odour (*r* = −0.76, *P* < 0.05) and the overall acceptability (*r* = −0.67, *P* < 0.05) of the gari have a significant negative correlation with the pH (Table 5). Also, the correlation of the sugar content of the gari was positive and significant with the acidity (*r* = 0.71, *P* < 0.05; Table 5). The effect of different processing centres on the sensory evaluation of gari as reported by Makanjuola *et al*. (2012) showed that the ash, lipid and fibre contents of gari have a negative correlation with all the sensory attributes evaluated (Table 5). The correlation of the data generated by Olaoye *et al*. (2015), on the effect of varieties and length of fermentation on the sensory evaluation of gari showed that the acceptability of the gari had a significant (*P* < 0.05) positive correlation with ash and fibre (*r* = 0.59), and the carbohydrate (*r* = 0.69) contents, but a negative correlation with moisture (*r* = −0.67, *P* < 0.05; Table 5). Also, the ash, fibre and carbohydrate contents of the gari have significant positive correlations with all the sensory attributes, and that of moisture content was negative for all the attributes. The protein content had a significant positive correlation with the gari appearance (*r* = 0.60, *P* < 0.05), and the cyanogenic potential content had a significant negative correlation with the aroma (*r* = −0.71, *P* < 0.05; Table 5). Oluwole *et al*. (2004) on their own part reported that the breakdown of starch in fresh cassava root by *Corynebacterium manihot* into simple sugars and the subsequent fermentation to produce lactic and formic acids resulting in pH drop could be linked to the production of the characteristics taste of gari. This correlation implies that breeders may use the relationship between the chemical composition of the cassava roots and the sensory acceptability of gari during the breeding process to improve on the varieties.

Apart from the relationship between the composition of the gari and its sensory attributes, the chemical composition of the cassava roots may affect the consumer preference of the gari/eba. This is because Akely *et al*. (2020) reported that the sweet taste of gari could be attributed to the relative high reducing sugar and low hydrogen cyanide contents of the cassava roots. Sanoussi *et al*. (2015) on their part observed that the dirty white colour of gari may be due to the high soluble sugars of the cassava root, which tends to caramelise during roasting. The texture of gari was also reported by Sanoussi *et al*. (2015) to be associated with the low fibres content of the cassava roots.

By and large, the primary sensory attributes that describe good gari are colour, taste, texture and aroma/flavour. Texture attributes of importance is crispiness in the case of dried gari, and hand feel before consumption in the case of eba. Taste attributes of importance are sweetness and sourness, and the combination thereof of the gari and eba. As a result of the relationship between the sensory attributes of gari and the carbohydrate (starch) and cyanogenic potential (CNP) contents of the raw roots, breeders should work more on increasing the starch and decreasing the CNP contents of the roots. Also, the gab in the texture of the gari/eba could be attributed to the differences in the production of gari from one location to another (Laya *et al*., 2018), thus, there is a need for further study to establish the different textural attributes of gari/eba based on locations.

## Conclusions

Substantial work has been published on the biochemical composition of fresh cassava roots and gari, on the postharvest processing to produce gari, including enrichment with additional ingredients (palm oil, soy flour, etc.) and on their effect on the sensory properties and perception by consumers of gari itself and gari reconstituted into eba. It is possible to correlate some biochemical or functional properties with specific sensory attributes, such as particle size with the perception of texture, or lactic acid content with the perception of sourness. These correlations make possible the evaluation of gari by instrumental means, to identify and select improved cassava varieties most suitable for transformation into gari, or inclusion as progenitors in breeding programmes. For further research on the interactions between consumer preferences assessments and the development of improved cassava varieties, the following areas currently have little or no information published and would benefit from more investigations: (i) comparison of the physical, functional and biochemical properties of gari produced from traditional and mechanised methods, with consideration of unit operation in order to identify critical control points and clarify the effect of the process on gari quality; (ii) the sensory attributes and consumer acceptability of gari produced from traditional and mechanised methods, and the sensory and instrumental texture profile analyses of eba from the same two methods; (iii) effect of postharvest preservation techniques of cassava roots such as ratooning and pruning on the processing ability, quality and sensory properties of gari and eba; (iv) effect of carotenoids biofortification on the quality and acceptability of eba, by comparing the properties and sensory attributes of eba made from white and yellow‐fleshed varieties.

## Author contributions

**Wasiu Awoyale:** Conceptualization (equal); Formal analysis (equal); Software (equal); Writing‐original draft (equal). **Emmanuel Oladeji Alamu:** Conceptualization (equal); Methodology (equal); Validation (equal); Visualization (equal); Writing‐review & editing (equal). **Ugo Chijioke:** Validation (equal); Visualization (equal); Writing‐review & editing (equal). **Thierry Tran:** Validation (equal); Visualization (equal); Writing‐review & editing (equal). **Noel Hubert Takam‐Tchuente:** Validation (equal); Visualization (equal); Writing‐review & editing (equal). **Robert Ndjouenkeu:** Validation (equal); Visualization (equal); Writing‐review & editing (equal). **Franklin Ngoualem Kegah:** Validation (equal); Visualization (equal); Writing‐review & editing (equal). **Bussie Maziya‐Dixon:** Conceptualization (equal); Funding acquisition (equal); Resources (equal); Writing‐review & editing (equal).

## Conflict of interest

The authors declare no conflict of interest in this work.

## Ethical approval

Ethics approval was not required for this research.

### Peer Review

The peer review history for this article is available at https://publons.com/publon/10.1111/ijfs.14867.

## Data Availability

Data sharing is not applicable to this article as no new data were created or analysed in this study.
